# Characteristics of Blood Vessels in Female Genital Schistosomiasis: Paving the Way for Objective Diagnostics at the Point of Care

**DOI:** 10.1371/journal.pntd.0004628

**Published:** 2016-04-13

**Authors:** Sigve Holmen, Hashini Nilushika Galappaththi-Arachchige, Elisabeth Kleppa, Pavitra Pillay, Thajasvarie Naicker, Myra Taylor, Mathias Onsrud, Eyrun Floerecke Kjetland, Fritz Albregtsen

**Affiliations:** 1 Norwegian Centre for Imported and Tropical Diseases, Department of Infectious Diseases Ullevaal, Oslo University Hospital, Oslo, Norway; 2 Institute of Clinical Medicine, University of Oslo, Oslo, Norway; 3 Department of Biomedical and Clinical Technology, Durban University of Technology, Durban, South Africa; 4 Optics and Imaging Centre, Nelson R Mandela School of Medicine, College of Health Sciences, University of KwaZulu-Natal, Durban, South Africa; 5 Discipline of Public Health Medicine, Nelson R Mandela School of Medicine, College of Health Sciences, University of KwaZulu-Natal, Durban, South Africa; 6 Department of Gynaecology, Oslo University Hospital, Oslo, Norway; 7 Department of Informatics, University of Oslo, Oslo, Norway; 8 Institute for Cancer Genetics and Informatics, Oslo University Hospital, Oslo, Norway; Universidade Federal de Minas Gerais, BRAZIL

## Abstract

**Background:**

The mucosal changes associated with female genital schistosomiasis (FGS) encompass abnormal blood vessels. These have been described as circular, reticular, branched, convoluted and having uneven calibre. However, these characteristics are subjective descriptions and it has not been explored which of them are specific to FGS.

**Methods:**

In colposcopic images of young women from a schistosomiasis endemic area, we performed computerised morphologic analyses of the cervical vasculature appearing on the mucosal surface. Study participants where the cervix was classified as normal served as negative controls, women with clinically diagnosed FGS and presence of typical abnormal blood vessels visible on the cervical surface served as positive cases. We also included women with cervical inflammatory conditions for reasons other than schistosomiasis. By automating morphological analyses, we explored circular configurations, vascular density, fractal dimensions and fractal lacunarity as parameters of interest.

**Results:**

We found that the blood vessels typical of FGS are characterised by the presence of circular configurations (p < 0.001), increased vascular density (p = 0.015) and increased local connected fractal dimensions (p = 0.071). Using these features, we were able to correctly classify 78% of the FGS-positive cases with an accuracy of 80%.

**Conclusions:**

The blood vessels typical of FGS have circular configurations, increased vascular density and increased local connected fractal dimensions. These specific morphological features could be used diagnostically. Combined with colourimetric analyses, this represents a step towards making a diagnostic tool for FGS based on computerised image analysis.

## Author Summary

Female genital schistosomiasis (FGS) is a disease of the female genitalia caused by a water-borne parasite. The parasite lays eggs that may be found in the genital organs and these eggs cause local inflammation and subsequent lesions. These lesions are called sandy patches and they are often accompanied by abnormal blood vessels. Sandy patches can be hard to see and clinicians have suggested that the typical blood vessels may serve as supporting or independent diagnostic indicators of FGS. Clinicians have described the specific features of the vessels as: appearing as circular shapes, forming a network and having branches. In this study, we examined the shape and appearance of cervical blood vessels by computer image analysis. We found that the blood vessels in women with FGS appear more often as circular shapes, at higher density and with more complex shapes compared to those in healthy women and in women with inflammation of the cervix for other reasons than FGS. Our findings suggest that cervical vessel appearance can contribute to the diagnosis of FGS. It could also be used in a future diagnostic tool using computer analysis. Further research is needed to determine if FGS can be diagnosed objectively.

## Introduction

The parasite *Schistosoma (S*.*) haematobium* deposits eggs in the urogenital tract causing urogenital schistosomiasis, and in females, it may cause the clinical syndrome known as female genital schistosomiasis (FGS) [1]. Studies have shown that 58–75% of women with detectable excretion of *S*. *haematobium* eggs in urine may have genital manifestations [1,2]. Likewise, in endemic areas, as many as 41% of women without detectable eggs in urine have been found to have genital lesions [1]. Genital lesions associated with *S*. *haematobium* ova are observed as grainy sandy patches, homogenous yellow sandy patches and rubbery papules [3,4]. In addition there is often the presence of abnormal blood vessels and occasionally these blood vessels are seen alone [1,3,4]. It has been hypothesized that the increased vascularity represents neovascularization [5]. However, a histopathological study on cervical biopsies did not find active neovascularization around schistosome eggs [6]. One case report presenting clinical images alongside the histological correlates showed dilated and tortuous venules containing viable schistosome eggs surrounded by a thrombus [7]. Clinically, these abnormal blood vessels have been described as being circular, reticulated, branched, convoluted and of uneven calibre [1,3,4].

FGS causes genital bleeding, pelvic discomfort and infertility [3,8]. There is also cross-sectional evidence that urogenital schistosomiasis increases the odds of having human immunodeficiency virus (HIV) by 2.9–4.0 times [9–11]. The diagnosis of FGS is based on the clinical identification of the characteristic genital lesions and abnormal blood vessels by visual inspection [12]. In areas where urogenital schistosomiasis is co-endemic with HIV, taking biopsies is of ethical concern due to the unnecessary risk of HIV transmission imposed on the patient by the iatrogenic lesion [3]. Urinary and genital egg excretion are poor proxy markers of lesions and systemic antigen / antibody tests do not provide information on the anatomical site of the morbidity [3]. Therefore, there is no adequate objective point-of-care diagnosis for FGS, making it necessary to explore alternative diagnostic tools to support the clinical diagnosis.

A safe, simple and reliable diagnosis for FGS is essential to provide patients with proper care and insight into this chronic condition [3]. Treatment for the active infection must be sought but the patient must also be made aware of the chronic nature of the lesions and the potential higher susceptibility to HIV. For many patients, it will be of enormous value to have an explanation for their genital symptoms and this may prevent repeated and unnecessary treatments for STIs based on a syndromic diagnosis.

Telemedicine is an approach that currently brings healthcare expertise to the point-of-care in the fields of pathology, dermatology and radiology [13]. We have previously suggested that the emerging smart phone availability in developing countries may be used as a platform to capture and interpret the characteristic sandy patches with computerized colour analyses as a possible approach to a simple diagnosis at the point of care [14,15]. Morphological vessel analysis may represent an additional approach to the diagnostic problem, either by itself or as a supplement to the colourimetric analysis. Such a diagnostic tool could be made freely available and easy to distribute to remote, rural areas as a software application, e.g. for smartphones or laptops. Many health clinics in low-resource settings already use digital cameras in their cervical cancer screening programs [16,17]. In these clinics it would not represent any additional cost to implement a free, software-based diagnostic tool for FGS detection.

We hypothesize that computerized morphological analyses may be used to objectively identify and quantify the abnormal blood vessels associated with FGS. In this study we explore the morphological features of cervical blood vessels observed in three groups: (1) in cases with FGS, (2) in endemic controls with other genital pathology and (3) in healthy endemic controls.

## Methods

### Ethics

The study was granted permission by the European Group on Ethics in Science and New Technologies (2011, Ref: IRSES-2010:269245), the Biomedical Research Ethics Administration, University of KwaZulu-Natal (March 2012, Ref: BF029/07), the regional Department of Health (DOH), Pietermaritzburg, KwaZulu-Natal (February 2009, Ref: HRKM010-08) and the Norwegian ethics committee, REC South East (2007 and 2011, Ref: 469-07066a1.2007.535), and the district Departments of Health and Education, KwaZulu-Natal in September 2012 and November 2012, respectively.

Participant details were kept confidential and each was allocated a unique number. All colposcopic images were non-identifiable: They only depict the uterine cervix and contain no names. All participants signed written, informed consent forms prior to investigation and were informed of the right to withdraw at any moment if they so wished. All participants were tested for human immunodeficiency virus, abnormal cytology and sexually transmitted infections. Test results were given to study participants who wanted their results. Counselling, referral and treatment were in accordance with standard DOH, South African guidelines. Anti retroviral therapy is free of charge in South Africa.

### Study population and area

The study participants were recruited as part of a larger study exploring FGS in young women in South Africa between 2011–2013. Participants were recruited from rural schools north and south of Durban, KwaZulu-Natal. These are areas endemic for urogenital schistosomiasis [18]. Sexually active, non-pregnant young women aged 16 and above were recruited. Recruitment was not based on symptoms or test results.

### Clinical investigation and questionnaire

Trained research assistants interviewed all participants in the local language (isiZulu). The questionnaire included questions on sexual behaviour, pregnancy and contraception. Gynaecological investigation was performed in those who consented. All clinical findings were recorded in electronic format along with a schematic representation of the lesion appearance, size and location (if present). As reported previously, images were captured colposcopically [14,15]. We searched the database (n = 1715) for images that fulfilled the following criteria: the cervix should be in the field of view, there should be no foreign material in the field of view (swab, spatula, acetic acid etc.) and the exposure and focus should be adequate for visualisation of the cervical surface and its features.

We defined three groups based on the clinician's evaluation and subsequent laboratory findings: (1) women with a normal appearing cervix and no schistosome egg excretion in urine, (2) women with abnormal blood vessels typical of FGS who had egg excretion in urine or presence of sandy patches and (3) women with a cervix presenting signs of inflammation (oedema, swelling and / or rubor) but no clinical findings typical of FGS and no schistosome egg excretion in urine. Each group consisted of randomly selected participants from the database.

### Laboratory analyses

A single urine sample was collected between 10 a.m. and 2 p.m. on the day of the clinical examination. Merthiolate-formalin solution (2%) was added to 10 mL of the sample. The sample was centrifuged and the pellet was deposited on two slides. Two independent technicians screened each of the slides by light microscopy [19].

Traditional Pap smears were done by the investigating clinician, preserved with a commercial cytological spray-fixative, then stained and analysed by cytotechnologists. Smears were reported using the Bethesda System of Reporting [20], and the categories of atypia included. Cellular atypia was classified as atypical squamous cells of unknown significance (ASCUS), low- and high-grade squamous cell intraepithelial lesions (LSIL and HSIL). The number of neutrophils and degree of inflammation were graded as none, mild, moderate or marked. A syndromic protocol was used to diagnose and treat findings at the point of investigation in alignment with the practice in rural clinics. Patients were contacted and asked if they had been treated and helped with further management of the disease once laboratory results were available. Patients with cellular atypia were referred to their local hospital for further management.

Cervico-vaginal lavage (CVL) was collected by spraying 10 mL of saline four times onto the ectocervical mucosa followed by withdrawal back into a syringe. The CVL was analysed for *Trichomonas vaginalis* using an in-house real-time PCR technique (Laboratory of Infection, Prevention and Control, University of KwaZulu-Natal, Durban, South Africa). *Chlamydia (C*.*) trachomatis* and *Neisseria gonorrhoea* were analysed using strand displacement assay on a ProbeTec machine (Becton, Dickinson and Company [BD], Franklin Lakes, New Jersey, USA). The CVL was also centrifuged, smeared on a slide and scored using the Nugent's criteria for bacterial vaginosis.

*Treponema pallidum* was analysed using rapid plasma reagin (RPR) on serum (Macro Vue 110, BD, Franklin Lakes, New Jersey, USA) and positive samples were confirmed using treponema pallidum haemagluttination assay (TPHA, Immutrep, Omega diagnostics Group PLC, Alva, United Kingdom). *Herpes simplex* type 2 antibodies were detected in serum using ELISA (Ridascreen HSV 2 IgG, Davies Diagnostics, Randburg, South Africa). HIV was detected in serum using a rapid antibody test (Core One Step HIV 1/2 test kit, Kendon laboratories, Durban, South Africa). Positive tests were confirmed using a different rapid antibody test (Sensa Tri-Line HIV Test Kit, Pantech (Pty) Ltd, Durban, South Africa).

### Blood vessel analyses

**Fig 1:**
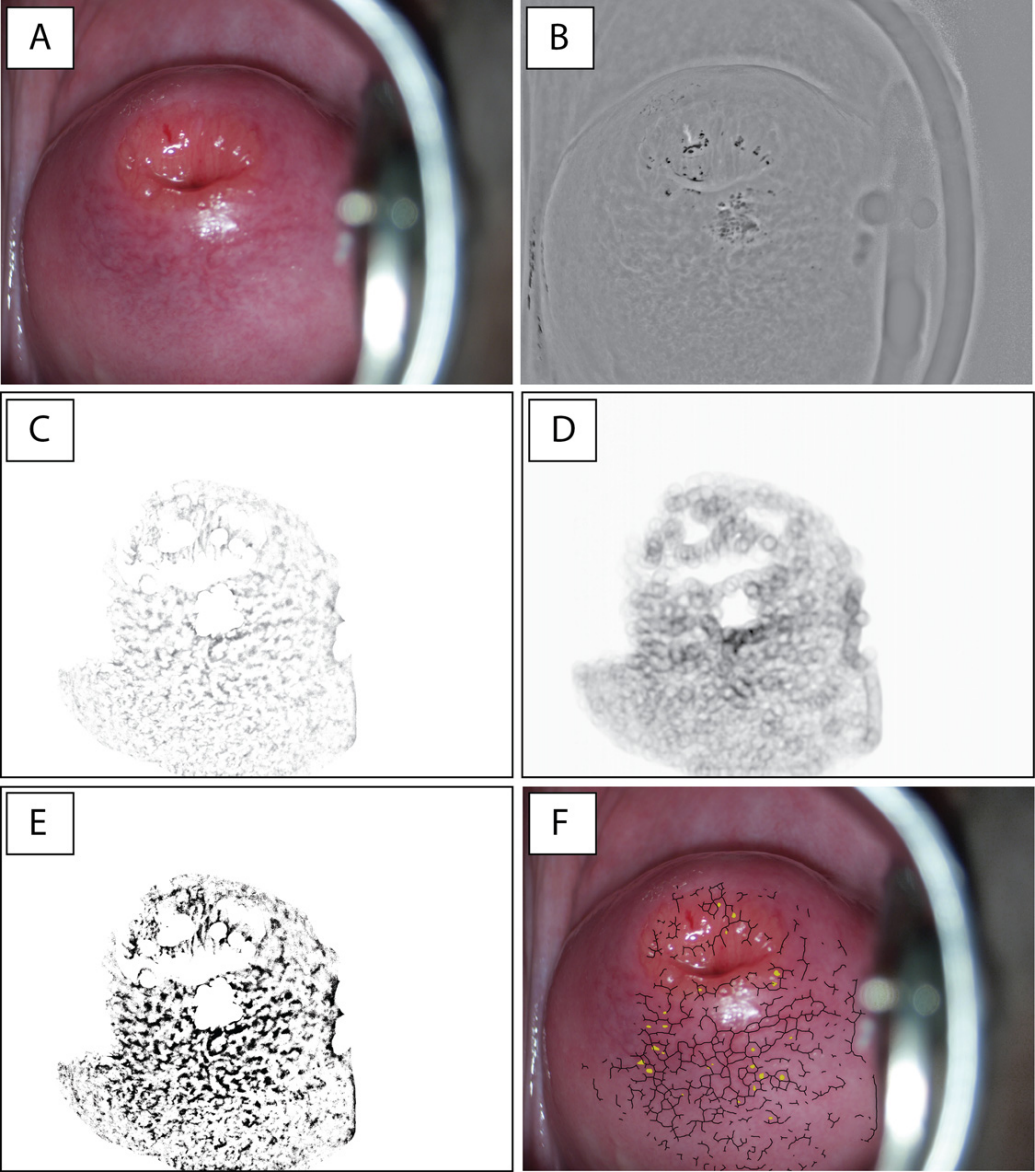
Image analysis of female genital schistosomiasis of the uterine cervix. **A.** The original colour image. Note that there is a light reflection at 6 o'clock and parts of the speculum can be seen on the right hand side (artefacts). These were removed automatically in the image processing. **B.** The product of multiplying the inverted "green channel" (from the "Red-Green-Blue" (RGB) colour space with the "saturation channel" (from the "Hue-Saturation-Value" (HSV) colour space). **C.** The region of interest (ROI) of image B with all pixels below the mean grey value removed. **D.** The result of convolution of the circular template and image C. The darker areas represent higher degrees of roundness. **E.** The result of adaptive local thresholding of image B using a modified Niblack method. **F.** The final output image automatically generated by the image analysis. It shows the ectocervix with numerous abnormal blood vessels (black skeleton) and the centres of circular structures identified by template matching (yellow dots).

All image processing and analyses were performed using ImageJ version 1.49 (open source, free software from National Institutes of Health, US). Plugins for ImageJ were written in Java (Oracle Corporation, Redwood Shores, US) to perform the specific analyses. A macro was written to automate the execution of all the analyses and to record all the results. Numeric results were recorded in a text file and images were generated to allow for visual verification of the analyses (an example is given in Fig 1F).

Colposcopic images often contain non-mucosal elements such as parts of the speculum, medical instruments and skin that should not be subject to the image analysis. As reported previously, we therefore applied automated detection of the region of interest (ROI) prior to analysis, using a previously described method, which identifies the cervix as the central area in the image having the highest values in the "a-channel" (of the "Lab colour space") [14].

In order to do morphological analyses on the cervical blood vessels, the blood vessel structures were identified by splitting the original colour image into the "green channel" (of the "RGB colour space") and the "saturation channel" (of the "HSV colour space"). The blood vessels, which appear bright red, have very low values of green and will therefore appear dark in the green channel. Furthermore, the blood vessels have high values of saturation and will therefore appear bright in the saturation channel. However, the ectocervix is convex and the surface is not evenly illuminated by the colposcope. It was therefore necessary to equalize the green and saturation channels. By generating an inverted image, which was subsequently smoothed using a Gaussian blur filter and then calculating the sum (addition) with the original image, the irregular illumination of the ectocervix was eliminated.

The product (multiplication) of the inverted green channel and the saturation channel resulted in an image where the blood vessels had higher values than the initial images (Fig 1B and S3 Fig). This resulting image was used for the subsequent analyses and is henceforth referred to as the processed image.

### Morphological analyses

Template matching allows for the identification of structures resembling a pre-defined template [21]. In ophthalmology, template matching algorithms have been developed to identify the optical nerve [21,22]. A circular template was generated to resemble the characteristic circular shape of the vessels as indicated by experienced clinicians (S6C Fig). For the template matching, the vascular structures were isolated from the processed image by removing all pixels below the mean value of grey (Fig 1C and S6B Fig). Template matching by convolution is a very processor-intensive process if applied as a pixel-by-pixel approach in the image domain, which would be impractical for a cell-phone application. Therefore, template matching was performed by converting the template and the image to the frequency domain by the fast Fourier transform [23], multiplying them and finally converting the result back to the image domain (multiplication in the frequency domain corresponds to convolution in the image domain). In the resulting image, areas with circular configurations have higher intensity (Fig 1D and S6D Fig). A threshold level was set to remove pixels below the 97.5th percentile of intensity (S6E Fig). The final image contains clustered pixels representing the centres of the matched circular structures (Fig 1D and S6F Fig). The number of circles identified was recorded for each image.

For the remaining morphological analyses, the processed image was converted to a binary image by an algorithm based on the Niblack [24] method for local adaptive thresholding, where the local threshold *T*(*x*, *y*) = *μ*(*x*, *y*) + *k* * *σ*(*x*, *y*), where μ and σ are the local mean value and standard deviation, respectively, within a sliding, circular window with a 50 pixel radius. Instead of a constant k-value determined empirically by trial-and-error [25] (S4E Fig), we inferred the optimal k-value for each image by first estimating the most likely distributions of foreground and background by using an expectation-maximisation (EM) algorithm (k-means clustering) (S4F Fig).

However, local adaptive thresholding may result in noise in areas with low contrast. We therefore only applied the Niblack threshold if the local standard deviation exceeded the mean standard deviation of all possible windows. If it did not, the area was defined as "low-contrast", and the threshold was set to the most likely foreground grey value (as estimated by the EM-algorithm). Furthermore, local adaptive thresholding may produce perimeter artefacts when applied on an isolated structure laid over a background, due to artificially high contrast between background and structure (S4E Fig). This was the case in our approach since we performed all analyses within a ROI. We therefore also used the most likely foreground grey value as threshold value for pixels whose window (50-pixel radius) fell within the perimeter of the ROI. Noise was removed from the resulting binary image by using a median filter with a radius of 4 pixels. An example of a resulting binary image is shown in Fig 1E.

After smoothing, the structures were skeletonised to a single pixel width, since in this context we are interested in the spatial configuration of the vessels. The skeletonisation was done using a lookup table to repeatedly remove pixels from the edges of objects in the binary image, analysing 3x3 pixel grids [26].

The skeletonised images were first used for calculating the distance between vessels and the total number of vessels per image. Distinct vessels were defined as not being connected by an 8-neighbourhood relationship. The distance between blood vessels was defined as the distance from any given vessel to its’ closest neighbour in a straight line. The skeletonised blood vessels were superimposed on the original colour image (Fig 1F) to allow for visual verification by the clinician. Experienced clinicians confirmed that the patterns portrayed in Fig 1F are recognized as typical in FGS cases (personal communication, EFK and HNGA).

Blood vessels may be considered fractals; objects whose details under magnification resemble the structure as a whole (S1 Text and S1 Fig and S2 Fig) [27]. The calculated fractal dimension of a network of vessels increases with increasing complexity [27]. A single point has a fractal dimension of zero, a straight line has a fractal dimension of one and a plane has a fractal dimension of two. However, a convoluted vessel in a plane will have non-integer fractal dimension (D) between that of a line (D = 1.0) and a plane (D = 2.0). Analysis of local connected fractal dimensions (LCFD) allows for identification of areas with higher or lower fractal dimensions within a fractal [27]. In ophthalmology, it was possible to identify patients with occlusion of the retinal artery by analysis of LCFD [27]. Another study found that patients with cerebral lacunar strokes had lower fractal dimensions of the retinal vessels compared to patients with minor cortical strokes [28].

Fractal lacunarity is a counterpart to the fractal dimension describing the heterogeneity of a fractal in terms of the size and distribution of holes or gaps [29]. If a fractal has large gaps or holes, it has high lacunarity. In gynaecology, fractal dimension and fractal lacunarity have been used to classify the uterine vessels seen in hysteroscopic images of patients with endometrial cancer and abnormal uterine bleeding [30].

We used the box counting method [29], with boxes doubling in size from 2–128 pixels, to estimate the general fractal dimension and lacunarity for the vascular network (S5 Fig). The LCFDs were calculated using the free ImageJ plugin FracLac version 2015Marb6206 (Charles Sturt University, New South Wales, Australia).

### Statistical analyses

Statistical analyses and graphs were produced using IBM SPSS Statistics Version 19 (IBM Company, Chicago, USA).

A sample size calculation showed that we needed a minimum of 36 cases in each group in order to be able to demonstrate a difference of 10% or more within a 95% interval of confidence if the standard deviation is 15 or less (arbitrary units). We decided to include 50 cases in each of the three groups.

Group characteristics were compared using the Kruskal-Wallis analysis of variance (age, days since last menstrual period and days since last intercourse) and the Chi-square test (pregnancy, hormonal contraception, STIs and findings in Pap-smear).

Comparisons of morphological characteristics were done using bivariate logistic regression; comparing the normal cervical appearance to those with blood vessels typical of FGS and those with cervical inflammation.

A multivariable logistic regression model was constructed using all the morphological characteristics and the group characteristics that differed significantly between the groups within an 85% interval of confidence. Variables were eliminated from the model one by one (backwards elimination) based on a minimum significance level of 0.15 and only if the model's likelihood ratio did not change significantly (p < 0.05). The final regression model was used to construct a ROC-curve to find the optimal cut-off value for identifying images with abnormal blood vessels typical of FGS, by identifying the point of the curve closest to the upper left corner. Classification accuracy was calculated using the cut-off value found on the ROC-curve.

## Results

### Group characteristics

The group characteristics are presented in Table 1. The women were similar in all respects except for the use of hormonal contraceptives, the prevalence of *C*. *trachomatis* and *Herpes simplex*. These variables were therefore considered when constructing the multivariable regression model. The morphological analyses presented in Table 2 show that women with FGS have significantly more circular blood vessels and a higher density of vessels are visible on the surface. In addition the fractal dimensions were higher in women with FGS.

**Table 1:** Group characteristics.

Variable	Abnormal blood vessels typical of FGS (n = 50)	Cervical pathology without signs of FGS (n = 50)	Normal cervical appearance (n = 50)	P-value of group difference
**Demographics and behaviour**				
Median age (range)	19 (16–26)	19 (16–26)	19 (16–27)	0.453^a^
Uses hormonal contraceptives	32%	56%	41%	**0.049**^b^
Has been pregnant	46%	60%	58%	0.315^b^
Median number of days since last period	17	15	15	0.581^a^
Median number of days since last intercourse	38	18	20	0.335^a^
Median age at sexual debut	16.5	17	16	0.856^a^
**Sexually transmitted infections**				
*Chlamydia trachomatis*	19%	40%	29%	**0.079**^b^
*Herpes simplex*	28%	45%	28%	**0.141**^b^
HIV	24%	34%	18%	0.190^b^
*Trichomonas vaginalis*	18%	31%	17%	0.699^b^
*Neisseria gonorrhoea*	4%	8%	6%	0.183^b^
*Treponema pallidum*	0%	2%	2%	0.589^b^
Median Nugent's criteria	7	8	8	0.237^a^
**Findings in Pap-smear**				
ASCUS^c^	25.9%	24.4%	24.0%	0.985^b^
LSIL^d^	37.0%	17.1%	28.0%	0.176^b^
HSIL^e^	3.7%	4.9%	0%	0.546^b^
Marked inflammation in Pap-smear	80%	82%	63%	0.182^b^
Marked neutrophilia in Pap-smear	60%	53%	36%	0.217^b^

a Kruskal-Wallis analysis of variance

b Chi-square test

c Atypical squamous cells of unknown significance

d Low-grade squamous cell intraepithelial lesion

e High-grade squamous cell intraepithelial lesion

P-values in bold indicate variables considered for the multivariable regression model (inclusion criteria p < 0.15)

**Table 2:** Characteristics of the blood vessels.

		Abnormal blood vessels typical of FGS (n = 50)	Cervical pathology^a^ without signs of FGS (n = 50)	Normal cervical appearance (n = 50)
		Median (range)	Median (range)	Median (range)
**Circularity**				
	Number of circular template matches^a^	20.5 (1–63) **	9.0 (0–29)	10.5 (0–23)
		OR 1.12 for an increase of 1 template match (95% CI: 1.08–1.23)		
**Vascular density**				
	Number of blood vessels^b^	109 (51–238) **	82.5 (6–174)	72 (6–123)
		OR 1.29 for an increase of 10 blood vessels (95% CI: 1.13–1.47)		
	Distance between blood vessels (px)	62.0 (49.9–98.2) *	71.9 (48.9–720.1)	84.0 (49.8–811.1)
		OR 0.96 for an increase of 1 px (95% CI: 0.93–0.99)		
**Fractal dimensions**				
	Fractal dimension^d^	1.029 (0.938–1.115) *	0.987 (0.817–1.074)	1.011 (0.820–1.086)
		OR 1.16 for an increase of 0.01 dimensions (95% CI: 1.04–1.29)		
	Fractal lacunarity^e^	0.206 (0.160–0.288) *	0.231 (0.180–0.344) *	0.221 (0.173–0.280)
		OR 0.83 for an increase of 0.01 in lacunarity (95% CI: 0.70–0.98)	OR 1.21 for an increase of 0.01 in lacunarity (95% CI: 1.04–1.42)	
	Mean local connected fractal dimension	1.011 (0.978–1.035)	1.010 (0.954–1.028)	1.008 (0.970–1.032)
	Peak local connected fractal dimension	1.501 (1.395–1.564) *	1.485 (1.265–1.563)	1.477 (1.268–1.580)
		OR 1.17 for an increase of 0.01 dimensions (95% CI: 1.05–1.31)		

a. Defined by the clinician as oedema, swelling and / or rubor.

b. Presence of configurations matching a predefined circular template

c. Absolute number of vessels per image

d. Structural complexity

e. Structural heterogeneity

* Results differ significantly from the normal cervical appearance, evaluated using bivariate logistic regression, p < 0.05

** Results differ significantly from the normal cervical appearance, evaluated using bivariate logistic regression, p < 0.001

OR (Odds ratio) is calculated in reference to the normal cervical appearance

**Fig 2:**
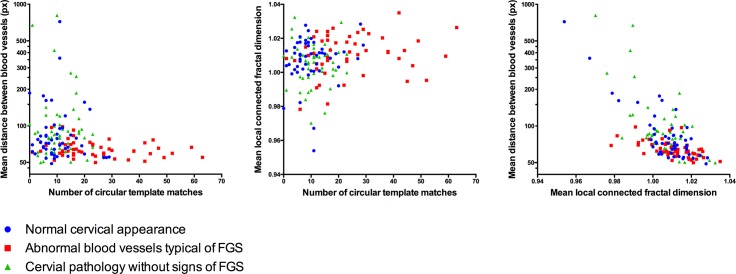
Scatterplots showing the co-variation of variables included in the regression model. The distribution of values of each of the variables included in the regression model differ between the women with FGS and the two other groups: The number of circular template matches is higher, the distance between vessels is lower and the mean local connected fractal dimension tends to be lower, although the latter is not significant.

In women with FGS, we found that the strongest morphological predictor of pathology was the presence of circular configurations in the vascular network (Table 3). We also found increased vascular density and increased vessel complexity (as estimated by LCFD) in women with FGS (Tables 2 and 3 and Fig 2).

**Table 3:** A multivariable regression model describing the association between abnormal blood vessels typical of FGS and morphological vessel characteristics.

Variable	Coefficient^a^	Odds ratio (95% CI)	p-value
Circular template matches	0.147	1.16 (1.08–1.25)	< 0.001
Mean distance between vessels	-0.042	0.96 (0.93–0.99)	0.015
Mean local connected fractal dimension	-0.531	0.590 (0.33–1.05)	0.071
Constant	54.464		

a. The regression coefficients were used to calculate the ROC curve (Fig 3) and the optimal cut-off value for a positive FGS diagnosis.

In the multivariable analysis, the number of blood vessels, general fractal dimension, general fractal lacunarity and peak LCFD were eliminated from the regression model along with the possible confounders; hormonal contraceptives, *C*. *trachomatis* and *Herpes simplex* (Table 3). The final regression model was used to generate a ROC curve (Fig 3) and the optimal cut-off value was -0.037. Using this cut-off value for the regression model, the model’s classification accuracy was calculated (Table 4). Finally, by defining the images with blood vessels typical of FGS as positive cases and the combined set of images with normal cervical appearance and cervical inflammation as negatives, the overall ability to classify the image correctly was 78% for positive cases and 80% for negative cases.

**Fig 3:**
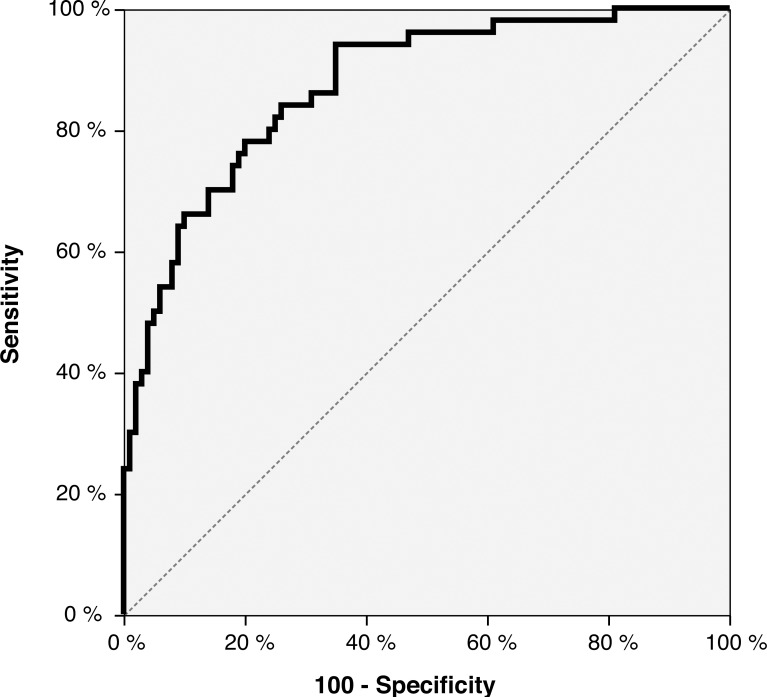
Receiver operating characteristics curve for the diagnosis of FGS by blood vessel analysis. The curve was generated using the regression model presented in Table 3. Area under the curve = 0.872.

**Table 4:** Classification of images using the proposed regression model.

Regression model^a^	FGS	Other cervical pathology	Normal cervix
Blood vessel^b^ negative	11 (22%)	38 (76%)	42 (84%)
Blood vessel^b^ positive	39 (78%)	12 (24%)	8 (16%)

^a^ Using a cut-off value of -0.037 for a positive diagnosis

^b^ Typical for FGS (circular, high density, with high fractal dimensions)

## Discussion

In this study we have demonstrated that computerised, morphological image analyses can be used to distinguish blood vessels typical of FGS from normal vessels and from blood vessels on cervices with signs of inflammation caused by other diseases. Using the morphological feature of circularity (as determined by template matching), distance between vessels and LCFD, we were able to classify the images with an accuracy of 78% for positive cases and 80% for negative cases.

### Clinical applicability

It has been reported that abnormal blood vessels typical of FGS present with specific morphological features [3]. Our findings support this observation and confirm that assessment of vessel appearance is of clinical significance when diagnosing FGS. Furthermore, our findings suggest that computerised image analyses of blood vessels visible on the cervical surface may play a role in developing a diagnostic tool for FGS, possibly together with colour analyses of sandy patches [14,15]. The World Health Organization recommends criteria for point-of-care tests: Affordable, Sensitive, Specific, User-friendly, Rapid and robust, Equipment-free and Deliverable to end-users (ASSURED) [31–33]. No such diagnostic tools exists for FGS and, to our knowledge, none are in the pipeline. Cellular phones are increasingly available in schistosomiasis endemic areas and a software-based diagnostic tool can be made freely available (for download) for use on existing devices such as smartphones, tablets or laptops. The software can be designed in a user friendly way and it can analyse the images instantly on the device, at the point of care (< 10 seconds using current laptops). However, it needs to be verified whether such a method is sufficiently accurate and robust when applied in a low-resource setting by local health professionals.

All the image analyses performed by this research group have been done using the Java-framework provided by ImageJ, which is open source and available on a number of platforms. It can therefore easily be deployed on any unit capable of running ImageJ. For implementation on Android devices, the necessary Java-libraries would simply need to be bundled in the application package. For other devices (not running Java), implementation might prove to be more laborious as the methods might need to be rewritten to conform to the operating system’s base language.

The current paper explores the possibility of using the morphological features of the abnormal blood vessels associated with FGS but this research group has previously published methods for diagnostics based on image analysis using colour to detect sandy patches [14,15]. The significance of these various lesions in terms of the pathophysiology and morbidity of FGS is not well understood. They may represent different aspects of the disease, different degrees of severity or progression. A diagnostic algorithm should include all the characteristic lesions and future studies may try to decipher their individual significance. Combined, they might also provide increased diagnostic accuracy [15].

We found a significantly higher number of circular features in the vascular network of patients with FGS. Furthermore, we found decreased distance between vessels and increased LCFD in women with FGS, indicating that the cervical vascular network is denser and morphologically more complex than in other diseases or in normal cervices. Increased LCFD is a morphological feature that requires computer analysis and will therefore only be clinically relevant when the diagnostic software has been introduced. However the two other findings (circular vessels and increased vessel density) can be recognised by a trained clinician and therefore represent objective, clinical observations that complement and support diagnosis of FGS that is based on inspection.

### Pathophysiological hypotheses

The circular vessel configuration which is seen more frequently on cervices of women with FGS could represent an area where an egg granuloma acts as a foreign body that locally prevents the growth of blood vessels or pushes them aside as the granuloma grows. Alternatively, the occlusion of a blood vessel by a schistosome worm-pair; its eggs and/or egg-induced thrombosis could cause dilatation and varicose-like distortion of the vessel [7].

In ophthalmology, the retinal vasculature has areas with increased LCFDs in patients with occlusion of the central vein or retinal artery [27]. Our finding of increased LCFDs in women with FGS therefore supports the argument that occlusion of small vessels may in fact represent an important feature in urogenital schistosomiasis.

### Limitations

We were not able to assess vessel calibre, as the images did not provide sufficient detail for this. Uneven calibre has been proposed as one of the characteristic features of FGS. However, this is difficult to assess without high magnification colposcopy and may therefore not be suitable for a low-cost, simple diagnostic approach.

Furthermore, the analyses in this study were performed on images acquired colposcopically. However, colposcopes are generally not available in clinics in endemic areas [34]. Previous attempts at performing computerised colour analyses on images of simulated low quality have shown good results [14]. Simple, handheld cameras have been evaluated for use in visual inspection with acetic acid (VIA) in cervical cancer screening programmes [35,36]. Similarly, this method should be evaluated on images acquired using simple devices such as handheld cameras or mobile devices. One obstacle that will need to be addressed when using simple handheld devices, is even illumination of the field. This is provided when using a colposcope, but for handheld devices it might be necessary to use an external light source such as a flashlight or a lamp. Furthermore, where colposcopes are available, these are primarily used for cancer screening. It would therefore be prudent not to depend on such equipment for the diagnosis of FGS, as there could be a false reassurance in regards to excluding cancer as a differential diagnosis if the colposcope is sometimes used for the diagnosis of FGS without targeted screening for cancerous lesions.

The analyses of colposcopic images are also limited by the narrow depth of field (the area in focus) provided by the colposcope. Since the ectocervix is generally convex in shape, only parts of its surface can be within the depth of field at once when photographed by a colposcope. Furthermore, it is rarely possible to visualise all the surfaces of the lower genital tract, especially the fornices and the vaginal wall. This may result in false negative cases in which lesions are missed. However, this would skew our results towards the null-hypothesis, thus strengthening our findings.

The participants in this study are young women aged 16–27 and cervical appearance may differ in older women due to transformation of the ectocervical columnar epithelium by metaplasia into squamous epithelium (the transformation zone) with increasing age [37].

### Conclusion

Blood vessels typical of FGS appear in circular configurations, have increased density and increased local connected fractal dimensions (LCFD). Future studies should assess these findings alongside the colourimetric analyses of the sandy patches in images acquired using a simple digital camera. Furthermore, the digital application should be explored as a supplement to the visual inspection.

## Supporting Information

S1 Text Fractals and blood vessel morphology. Details about fractals and how we analyses blood vessel morphology using fractal properties.
(DOCX)

S1 Fig South Africa's coastline can be considered a fractal. South Africa's coast line reveals more details when visualised at increasing magnifications (from left to right) but the structures are fundamentally similar.
(TIF)

S2 Fig Measuring the outline of South Africa. The outline of South Africa can be measured with increasing levels of detail (number of line segments), revealing a more complex structure and also adding to the length of the outline.
(TIF)

S3 Fig Combining the green and saturation channels. The multiplication of the inverted green channel and the saturation channel results in an image where the blood vessels have "boosted" values, appearing more clearly than in either of the two original colour channels.
(TIF)

S4 Fig Thresholding. **A.** Original colour image. **B.** Equalized product of the inverted Green channel and the Saturation channel. The automatically detected region of interest (ROI) is indicated in red. **C.** The standard deviation of grey values in a 50px window calculated for each pixel and represented as relative levels of grey values (0–255). **D.** Thresholding performed using the mean grey value. **E.** Thresholding using the standard Niblack method with a k-value of -0.2. Notice the perimeter artefact as a thick white line around the ROI. **F.** Thresholding using Niblack's method with calculation of optimal k-value per 50px sliding window and elimination of the perimeter effect.
(TIF)

S5 Fig Box counting. **Top**: A binary blood vessel structure after image processing. **Middle**: A log-log plot showing the number of boxes (N) required to cover the blood vessel in relation to box size (ε). The slope of the dashed red regression line is 1.315, corresponding to the estimated fractal dimension, D. **Bottom:** A log-log plot showing the mean λ-value in relation to decreasing box size (ε). The slope of the dashed red regression line is 0.454, corresponding to the estimated lacunarity of the blood vessel.
(TIF)

S6 Fig Template matching. **A.** The original colour image. **B.** After extracting the region of interest, the boosted image is thresholded by using the mean grey value. This leaves primarily blood vessel structures in the image. **C.** The circular convolution template. **D.** The result of the convolution of B and C (multiplication in the frequency domain). **E.** The result of thresholding image D on the 97.5th percentile of pixel value. **F**. The result of the template matching (E) superimposed on the original colour image (A).
(TIF)
