# Supplementary material for: Characteristics of Blood Vessels in Female Genital Schistosomiasis: Paving the Way for Objective Diagnostics at the Point of Care
Source: PLoS Negl Trop Dis. 2016 Apr 13;10(4):e0004628. doi: 10.1371/journal.pntd.0004628 (PMC4830560; doi:10.1371/journal.pntd.0004628)
Supplement: S1 Text — Details about fractals and how we analyses blood vessel morphology using fractal properties. (DOCX) [file pntd.0004628.s001.docx]

# S1 Text. Fractals and blood vessel morphology

## What is a fractal?

Fractals are objects whose details under magnification resemble the structure as a whole (Landini et al. 1995). This is true for a large number of naturally occurring phenomena (Mandelbrot 1983). By increasing the magnification of the structure, new details are revealed, but always resembling the structure as a whole. Let's consider the example of South Africa's coastline. As we increase the magnification, we discover new details of the coastline but every image is still a map showing land, coast and sea (S1 Fig). The coastline is self-resembling at increasing levels of magnification and may therefore be called a fractal.

## Blood vessels as fractals

Equal to the coastline of South Africa, magnification of blood vessels reveals self-similar structures. Arteries branch off the aorta, arterioles branch off the small arteries and finally capillaries branch off the arterioles. At each level of magnification the blood vessel structure is similar. Increased branching, reticularity and convolution are all factors representing increased morphological complexity. The fractal dimension is an expression of complexity as a ratio of change in detail to change in scale.

Let's consider the example of South Africa's coastline again. Seen from a blurry satellite image we can attempt to measure the length of the coastline (S2 Fig, left). However, using more detailed images, such as from aerial photographs, we could measure the coastline in greater detail, adding small bays and spits to the outline, representing a more complex structure. However, this also results in the measured length of the coastline to increase (S2 Fig, middle and right). In a theoretical attempt at getting an even more detailed measurement of the coastline, we could measure it by walking along the coastline using a small ruler or even using a microscope. This would add the edges of every grain of sand and pebble to the measurement of the coastline, resulting in an even more complex and longer coastline.

The relationship between the increase in magnification and the increase in complexity (as measured by length in the example of the coastline) can be used to express the complexity of a fractal through the notion of fractal dimension. A fractal dimension of one means that, as the scale with which the fractal is observed increases, the level of detail remains exactly the same. This is the case for a straight line: regardless of the scale, it will still remain a straight line.

## Isolating the blood vessels

In order to do morphological analyses on the cervical blood vessels, the blood vessel structures had to be isolated. It is well known from photocolposcopy that the application of a green filter on the light source may render blood vessels easier to see because they appear almost black as they reflect very little green light (Mayeaux & Cox 2011). Likewise, the vessels appear very dark in the Green channel in a regular RGB colour image acquired in normal (white) light. Furthermore, the blood vessels appear in a strong red colour, representing high values in the Saturation channel of the HSV colour space. It was therefore possible to use two different colour channels for the isolation of cervical blood vessels.

However, the ectocervix has a convex shape, reflecting the light unevenly across the surface. It was therefore necessary to equalize the colour channels before analysis. This was done by generating an inverted image for each channel, which was subsequently smoothed using a Gaussian blur filter and then calculating the sum (addition) with the original image. The inverted Green channel could then be multiplied with the saturation channel to produce an image with "boosted" values of the pixels with low values of green and high values of saturation (S3 Fig). This image is henceforth referred to as the boosted image.

## Thresholding

The product obtained from the multiplication of the inverted Green channel and the Saturation channel is still not suitable for fractal analyses. The blood vessels need to be represented in a binary image with only two colour values; black and white (0 and 1). This can be achieved by defining a threshold value, T, of grey-level (E1). This is known as thresholding.

|  | $pixel =\left( pixel \geq T \right) ? object : background$ | (E1) |
| --- | --- | --- |

However, determining an optimal threshold value that works in a large number of images obtained from different cameras under different conditions is not trivial. It is therefore more useful to calculate an adaptive threshold per image, such as using the mean grey value (S4 Fig, D). Although we equalized the colour channels used to generate the boosted image, there will always be local variations in illumination and contrast within an image. Therefore, we applied the local adaptive thresholding Niblack method (Niblack 1986) to ensure optimal thresholding of each pixel (S4 Fig, E). It is applied per pixel using the mean (µ) and standard deviation (σ) calculated within a window surrounding each pixel (E2).

|  | $pixel =\left( pixel \geq\mu+ k * \sigma\right) ? object : background$ | (E2) |
| --- | --- | --- |

The k-value (E2) represents a constant of which the sign (positive or negative) determines whether to keep dark or light objects and the magnitude determines how "strict" the thresholding should be. This can be set to a default value of -0.2 or 0.2 depending on whether the background is dark or light (Sezgin & Sankur 2004). Otherwise it can be found empirically by trial and error (Chaki et al. 2014). Neither of these approaches will yield an optimal k-value. However, it is possible to determine the optimal k-value if the distributions of foreground and background pixels are known. These can be estimated by expectation-maximization (EM), which is an iterative algorithm to find distributions with the highest likelihood (Redner & Walker 1982). The k-value can then be expressed as:

|  | $k =\frac{d\left( \frac{1}{2}-F \right)-\frac{1}{d}ln\left( \frac{F}{1-F} \right)}{\sqrt{1-d^{2}}\left( F-1 \right)F}$ | (E3) |
| --- | --- | --- |

Where d is the number of standard deviations (σ) separating the mean background and foreground values and F is the probability of the foreground distribution.

Local adaptive thresholding techniques have a tendency to generate noise in areas with low contrast. This has been solved in a number of ways, such as Bernsen's method (Sezgin & Sankur 2004) of setting a lower threshold value (lc_t_) of local contrast (lc) below which the threshold value will default to 128 (E4). Local contrast (lc) is defined as the difference between maximum and minimum grey values within a sliding window.

|  | $\begin{matrix} if \left( lc < {lc}_{t} \right) pixel = \left( midgrey \geq128 \right) ? object : background \\ else pixel = (pixel \geq midgrey) ? object : background \end{matrix}$ | (E4) |
| --- | --- | --- |

We used a similar approach, but instead of evaluating the local contrast ad modum Bernsen, we evaluated the local standard deviation (σ) of grey values in reference to the mean standard deviation ($\bar{\sigma}$) within the ROI as a more robust measure of contrast (E5). If the contrast was low, Niblack's method was not applied and the threshold value was set to the estimated mean foreground value (µ_F_).

|  | $\begin{matrix} if \left( \sigma< \bar{\sigma} \right) pixel = \left( {pixel \geq\mu}_{F} \right) ? object : background \\ else pixel = \left( pixel \geq\mu+ k * \sigma\right) ? object : background \end{matrix}$ | (E5) |
| --- | --- | --- |

Furthermore, the Niblack method has an inherent flaw when working within a ROI. If the standard deviation within a sliding window is very high, as it will be close to the perimeter of the ROI (S4 Fig, C), the resulting threshold value will be either very low or very high, producing a perimeter artefact (S4 Fig, E). This can be avoided by extending the abovementioned procedure to include windows with very high contrast; exceeding the 97.5th percentile of contrast (E6). The result is elimination of the perimeter artefact (S4 Fig, F).

| $\begin{matrix} if \left( \sigma< \bar{\sigma} \vert\vert\sigma> \bar{\sigma} + 1.96 * \bar{\sigma}_{\sigma} \right) pixel = \left( {pixel \geq\mu}_{F} \right) ? object : background \\ else pixel = \left( pixel \geq\mu+ k * \sigma\right) ? object : background \end{matrix}$ | (E6) |
| --- | --- |

## Reticular, branched and convoluted

Blood vessels may be considered fractals; objects whose details under magnification resemble the structure as a whole (Landini et al. 1995). Increased branching, reticularity and convolution are all factors representing increased morphological complexity. The complexity of fractals may be estimated by calculating the fractal dimension, either of the entire fractal or locally as local connected fractal dimensions (LCFDs) (Landini et al. 1995). The fractal dimension is an expression of complexity as a ratio of change in detail to change in scale. E.g. a fractal dimension of one means that, as the scale with which the fractal is observed increases, the level of detail remains exactly the same. This is the case for a straight line: regardless of the scale, it will still remain a straight line.

To estimate the blood vessels' fractal dimensions we used a simple method that consists of estimating the above-mentioned scaling phenomenon by counting the number of rectangular boxes required to cover the fractal (Smith et al. 1996). By gradually decreasing the box size, more boxes are required to cover the fractal. However, if the fractal has a high degree of complexity, the number of boxes required will increase more with scaling than it would for a fractal of less complexity, thus representing a higher fractal dimension, D (S5 Fig). This can be expressed as the log-log relationship between the numbers of boxes, N, divided by the inverse box size, ε (E7).

|  | $D = \lim_{\varepsilon\to0} \frac{\log N_{\varepsilon}}{\log\left( 1/\varepsilon\right)}$ | (E7) |
| --- | --- | --- |

Lacunarity is another characteristic of fractals that describes their complexity in terms of heterogeneity. A fractal with many gaps and spaces is said to have high lacunarity. This can also be estimated by using the box counting algorithm (Smith et al. 1996). However, for lacunarity, the interesting measure, denoted λ, is the variation in number of pixels present from box to box. This can be expressed as the standard deviation (σ) divided by the mean (μ) number of pixels per box (E8).

|  | $\lambda= \left( \frac{\sigma}{\mu} \right)^{2}$ | (E8) |
| --- | --- | --- |

Using a sliding window, it is therefore possible to calculate a λ-value per pixel for each of the box sizes (ε). The lacunarity of the fractal can be expressed as the log-log relationship between the mean λ-value and box size, ε (S5 Fig, bottom).

## Circular features

The abnormal blood vessels typical of FGS have been described as having circular features (Kjetland et al. 2012; Kjetland et al. 2005; Norseth et al. 2014). The vascular structures in the boosted image (Figure 3) were isolated by removing all pixels below the mean grey level (S6 Fig, B). A circular template was generated to resemble the characteristic circular shape of the vessels as indicated by the clinicians (S6 Fig, C). The circular template was blurred to allow for a wider range of circular sizes. Template matching is a very processor-intensive process if applied as a pixel-by-pixel approach in the space domain. With mobile applications in developing countries in mind, we sought to find a less processor intensive method that performed adequately. Multiplication in the frequency domain corresponds to convolution in the space domain but multiplication is a much simpler operation. Therefore, template matching was performed by converting the template and the processed image to the frequency domain by the fast Fourier transform, multiplying them and finally converting the result back to the space domain (S6 Fig, D). In the resulting image, areas with circular configurations have higher intensity due to the intersection of convoluted circles. A threshold level was set to remove pixels below the 97.5th percentile of intensity. The final image contains clustered pixels representing the centres of the matched circular structures (S6 Fig, E). The number of circles identified was recorded for each image.
