# Supplementary figures and images for: Characteristics of Blood Vessels in Female Genital Schistosomiasis: Paving the Way for Objective Diagnostics at the Point of Care
Source: PLoS Negl Trop Dis. 2016 Apr 13;10(4):e0004628. doi: 10.1371/journal.pntd.0004628 (PMC4830560; doi:10.1371/journal.pntd.0004628)

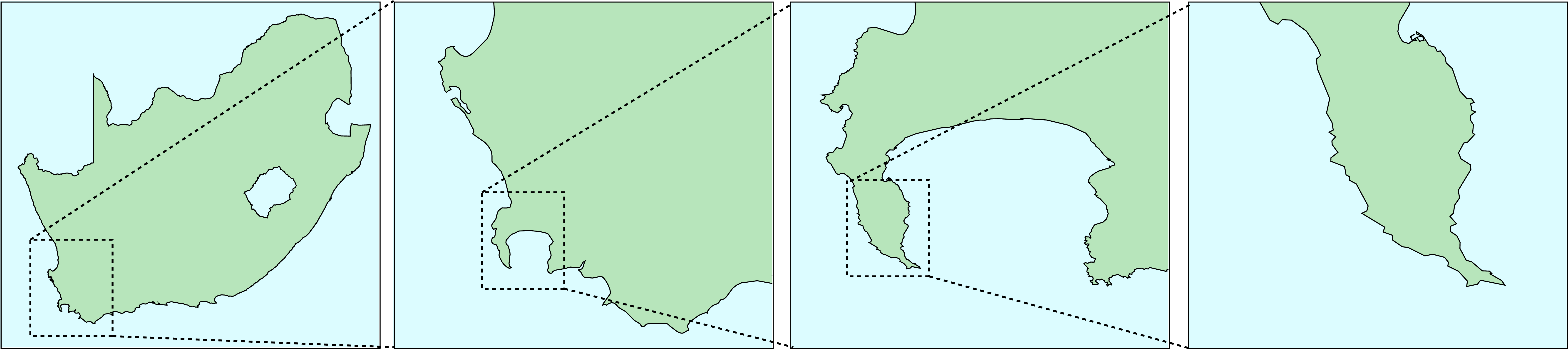

Supplement: S1 Fig — South Africa's coast line reveals more details when visualised at increasing magnifications (from left to right) but the structures are fundamentally similar. (TIF) [file pntd.0004628.s002.tif]

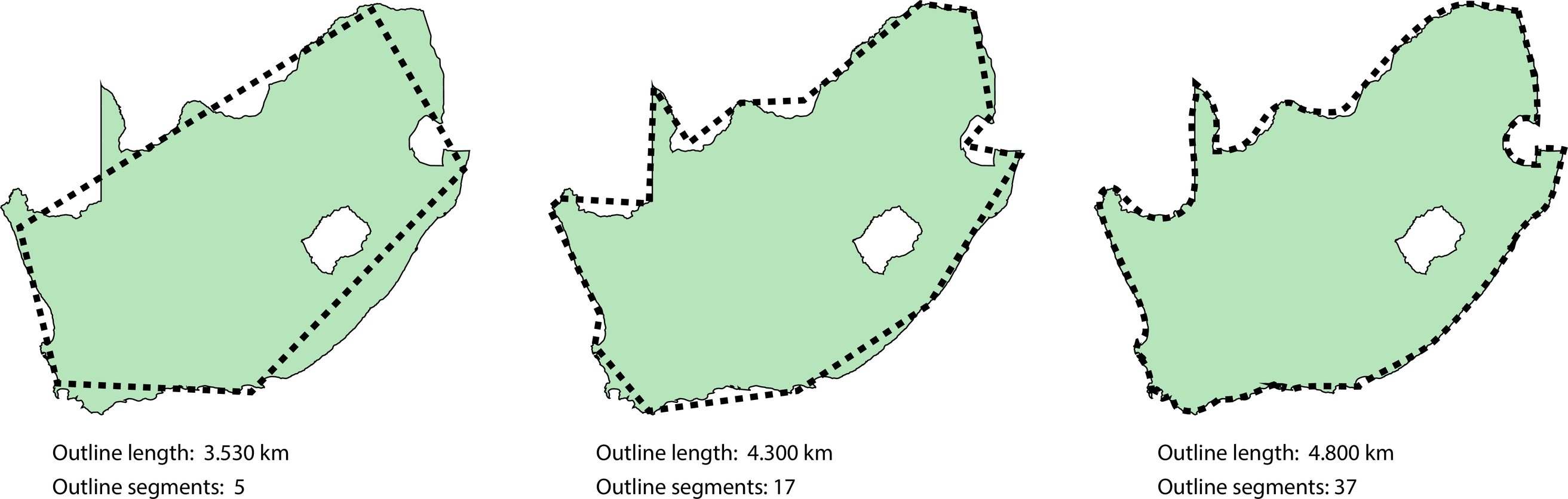

Supplement: S2 Fig — The outline of South Africa can be measured with increasing levels of detail (number of line segments), revealing a more complex structure and also adding to the length of the outline. (TIF) [file pntd.0004628.s003.tif]

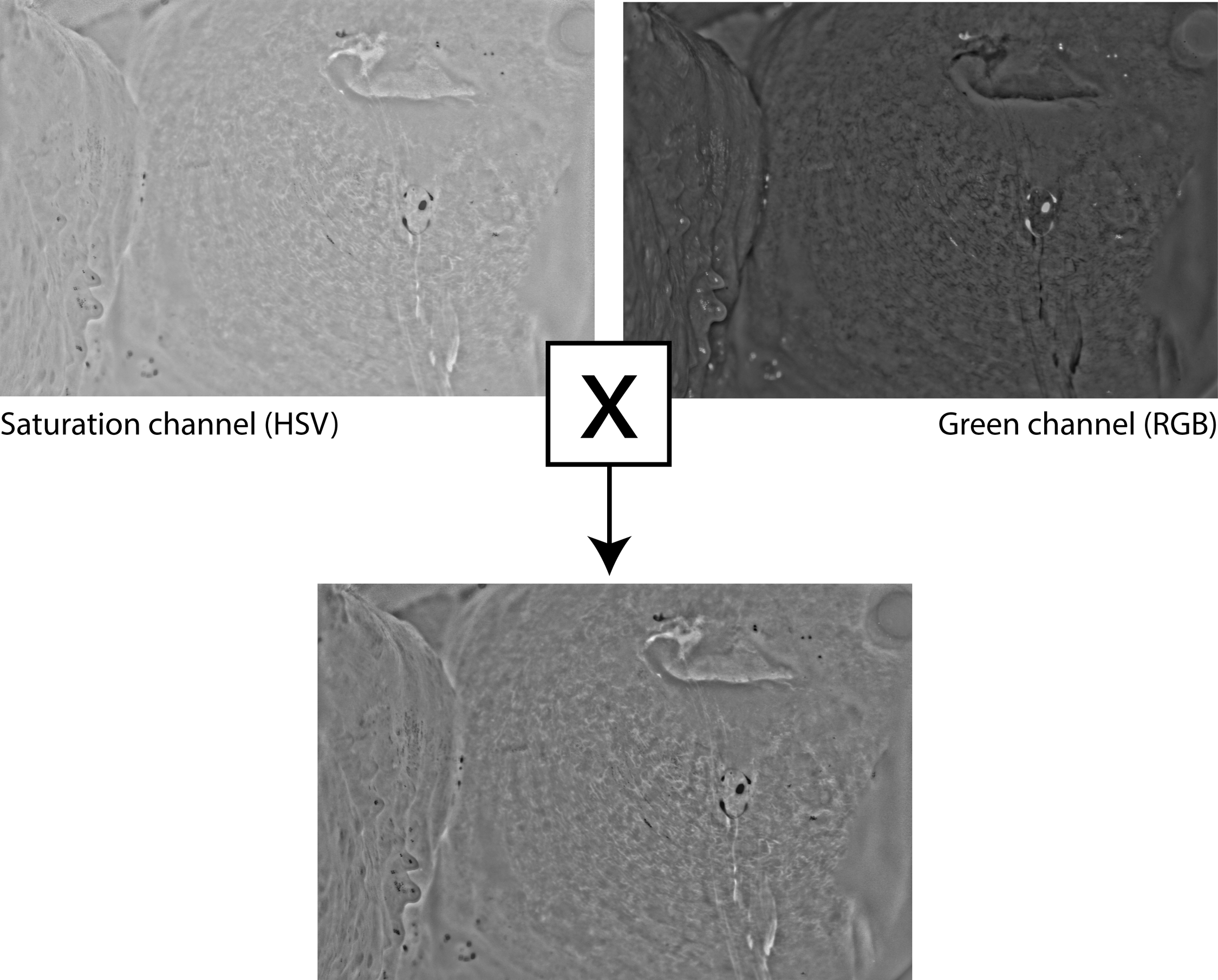

Supplement: S3 Fig — The multiplication of the inverted green channel and the saturation channel results in an image where the blood vessels have "boosted" values, appearing more clearly than in either of the two original colour channels. (TIF) [file pntd.0004628.s004.tif]

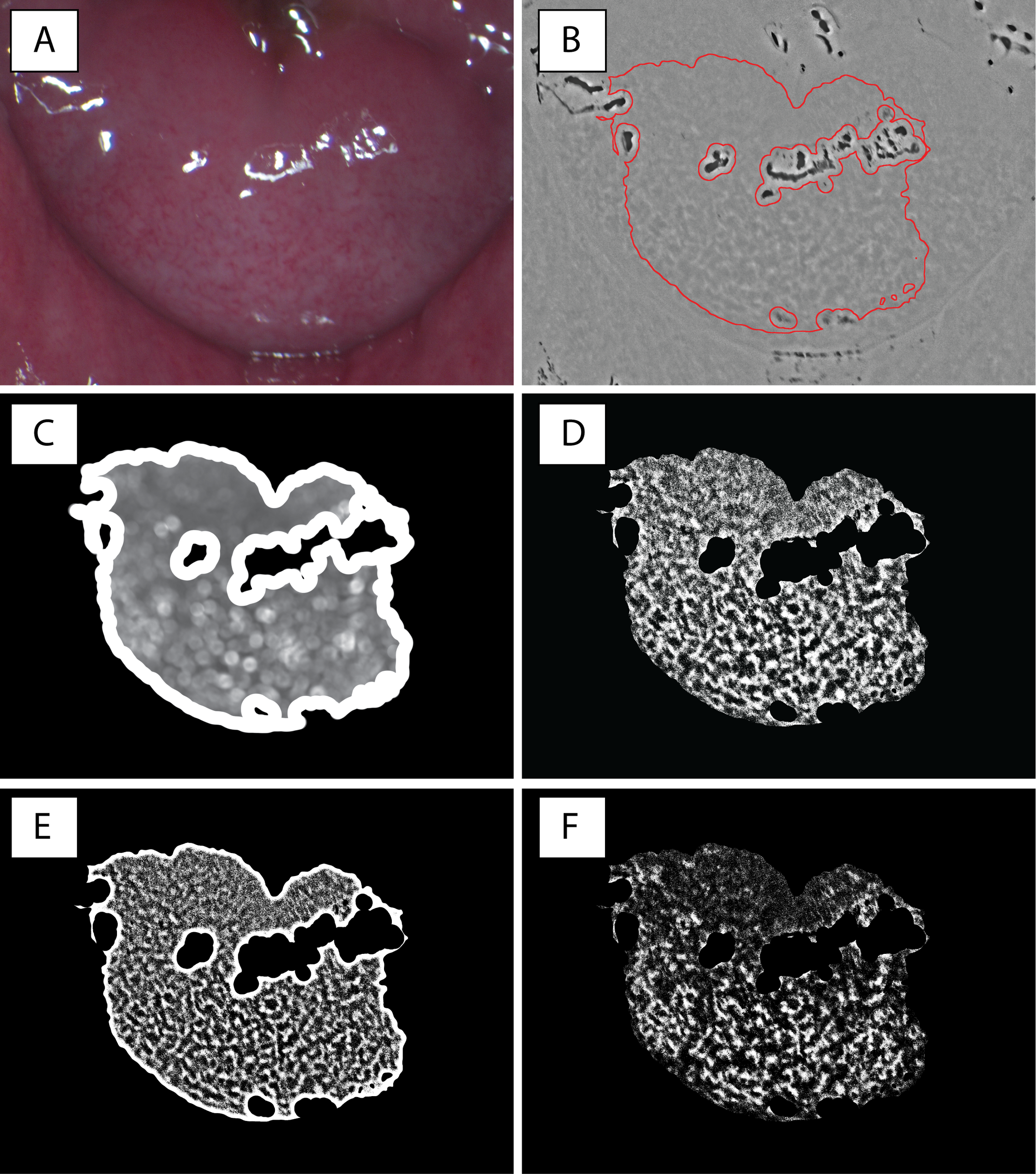

Supplement: S4 Fig — A. Original colour image. B. Equalized product of the inverted Green channel and the Saturation channel. The automatically detected region of interest (ROI) is indicated in red. C. The standard deviation of grey values in a 50px window calculated for each pixel and represented as relative levels of grey values (0–255). D. Thresholding performed using the mean grey value. E. Thresholding using the standard Niblack method with a k-value of -0.2. Notice the perimeter artefact as a thick white line around the ROI. F. Thresholding using Niblack's method with calculation of optimal k-value per 50px sliding window and elimination of the perimeter effect. (TIF) [file pntd.0004628.s005.tif]

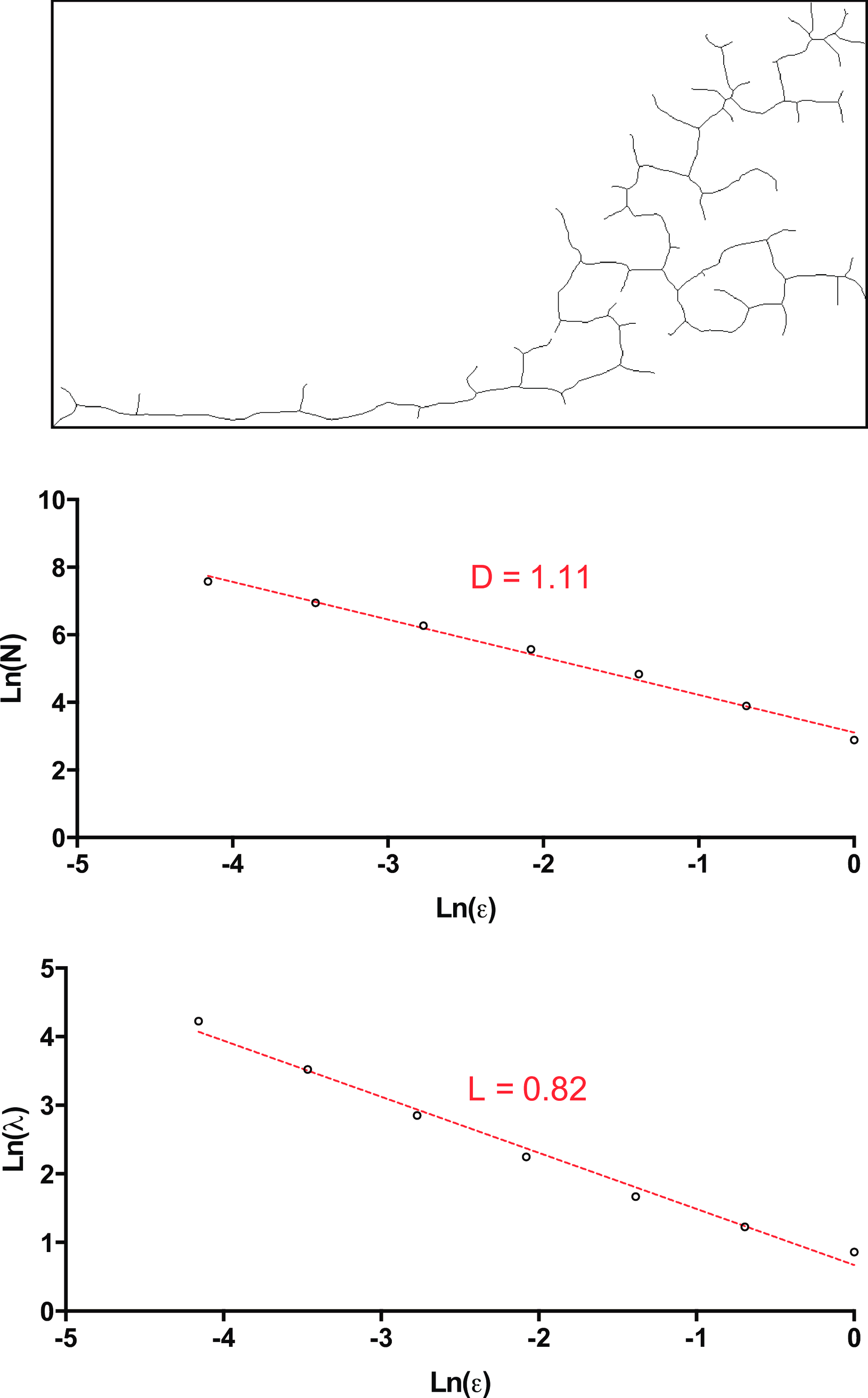

Supplement: S5 Fig — Top: A binary blood vessel structure after image processing. Middle: A log-log plot showing the number of boxes (N) required to cover the blood vessel in relation to box size (ε). The slope of the dashed red regression line is 1.315, corresponding to the estimated fractal dimension, D. Bottom: A log-log plot showing the mean λ-value in relation to decreasing box size (ε). The slope of the dashed red regression line is 0.454, corresponding to the estimated lacunarity of the blood vessel. (TIF) [file pntd.0004628.s006.tif]

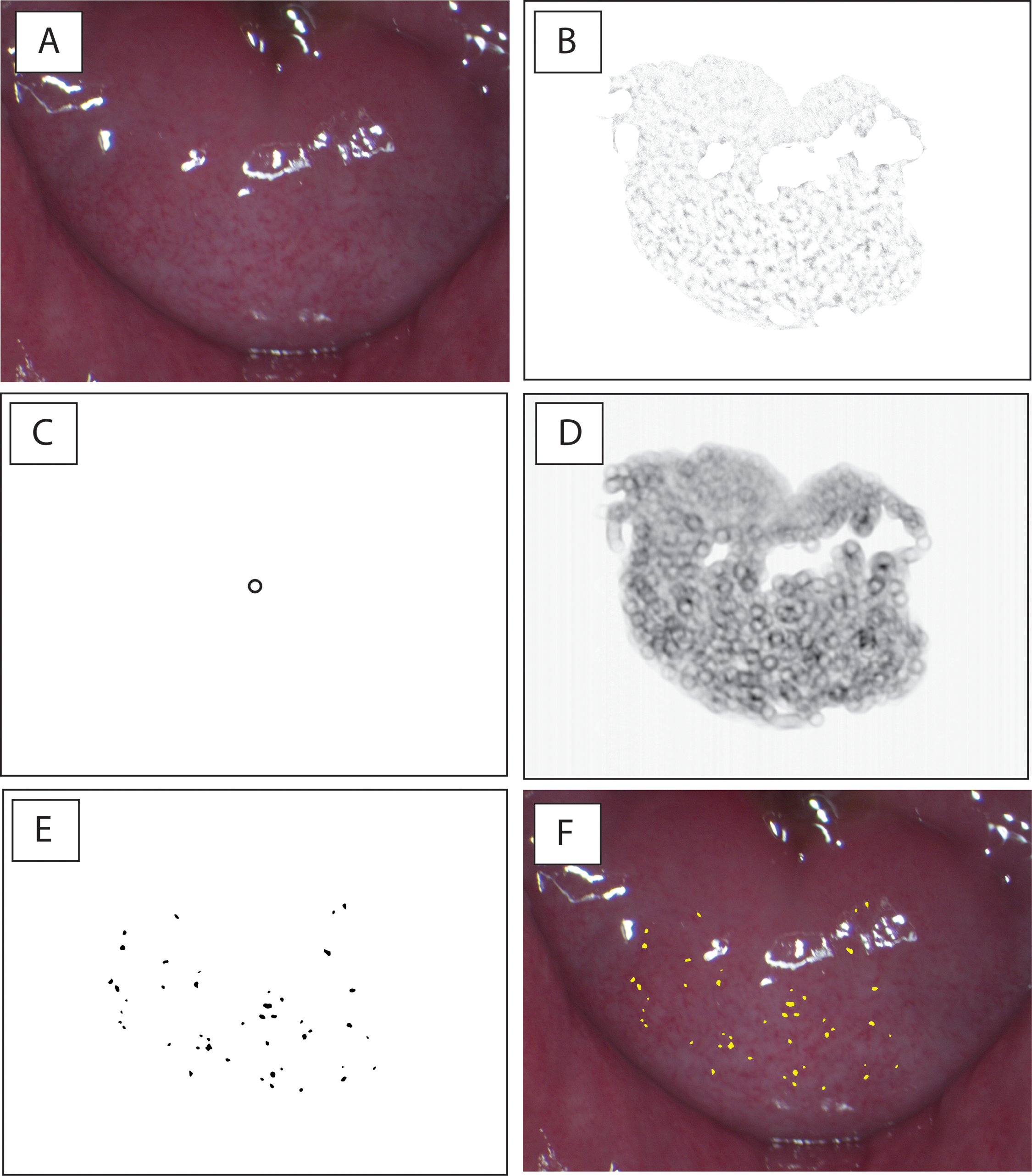

Supplement: S6 Fig — A. The original colour image. B. After extracting the region of interest, the boosted image is thresholded by using the mean grey value. This leaves primarily blood vessel structures in the image. C. The circular convolution template. D. The result of the convolution of B and C (multiplication in the frequency domain). E. The result of thresholding image D on the 97.5th percentile of pixel value. F. The result of the template matching (E) superimposed on the original colour image (A). (TIF) [file pntd.0004628.s007.tif]
